# A Case of Fluoroquinolone-Resistant Leprosy Discovered after 9 Years of Misdiagnosis

**DOI:** 10.1155/2016/4632369

**Published:** 2016-08-07

**Authors:** Onivola Raharolahy, Lala S. Ramarozatovo, Irina M. Ranaivo, Fandresena A. Sendrasoa, Malalaniaina Andrianarison, Mala Rakoto Andrianarivelo, Emmanuelle Cambau, Fahafahantsoa Rapelanoro Rabenja

**Affiliations:** ^1^USFR Dermatologie, Centre Hospitalier Universitaire Joseph Raseta Befelatanana, 101 Antananarivo, Madagascar; ^2^Centre d'Infectiologie Charles Mérieux, Université d'Antananarivo, 101 Antananarivo, Madagascar; ^3^APHP, Hôpital Lariboisière, Bactériologie, Centre National de Référence des Mycobactéries et de la Résistance des Mycobactéries aux Antituberculeux, 75475 Paris Cedex 10, France

## Abstract

We report a case of misdiagnosed leprosy in a 21-year-old Malagasy male, who, improperly treated, developed secondary mycobacterial resistance to fluoroquinolone. The patient contracted the infection 9 years prior to the current consultation, displaying on the right thigh a single papulonodular lesion, which progressively spread to the lower leg, back, and face. Initial administration of ciprofloxacin and prednisolone led to temporary and fluctuating improvement. Subsequent long-term self-medication with ciprofloxacin and corticosteroid did not heal the foul and nonhealing ulcers on the legs and under the right sole. Histopathological findings were compatible with lepromatous leprosy. Skin biopsy was positive for acid-fast bacilli and PCR assay confirmed the presence of a fluoroquinolone-resistant strain of* Mycobacterium leprae* (*gyrA* A91V). After 6 months of standard regimen with rifampicin, clofazimine, and dapsone, clinical outcome significantly improved. Clinical characteristics and possible epidemiological implications are discussed.

## 1. Introduction

Leprosy is a chronic infectious disease caused by* Mycobacterium leprae* that commonly affects skin and peripheral nerves. The diagnosis of leprosy is not always easy due to the great diversity of clinical manifestations, which depend mainly on the patient's cellular immunity to* Mycobacteria* [1], but also on his immune status and genetic factors [2]. Whereas the prevalence of leprosy has been significantly reduced in most endemic areas, its incidence has remained steady for the past decade. In Madagascar, where leprosy is considered a major public health concern [3], more than 1,000 new cases were reported annually from 2005 to 2014. Yet there has been progress in the worldwide fight against leprosy ever since WHO promoted multidrug therapy for multibacillary and paucibacillary leprosy. Major challenges still remain including the emergence of drug-resistant strains of* M. leprae*. In this report, we describe a case of misdiagnosed typical lepromatous leprosy caused by a secondary fluoroquinolone-resistant strain in a patient with probable drug-induced immunosuppression.

## 2. Case Report

A 21-year-old Malagasy male student, with no significant medical history, reported to our Dermatology Ward in May 2015, with extensive papulonodular lesions and nonhealing ulcers on his lower limbs. The patient had contracted the infection 9 years earlier, initially displaying on his right thigh a single papulonodular lesion, which progressively spread to his arms and legs and to his back and face within 5 years. Living in a remote area, the patient sought medical attention in a small private health centre. He was prescribed ciprofloxacin 1 g per day associated with prednisolone 10 mg per day for one week. This treatment resulted in moderate yet fluctuating improvement before the patient presented foul and nonhealing ulcers on his legs and under his right sole, as well as recurrent hemorrhagic rhinitis. This was followed from 2001 on by long-term self-administration of ciprofloxacin 1 g per day and prednisolone 10 mg per day, as well as topical eosin and betamethasone ointment.

**Figure 1:**
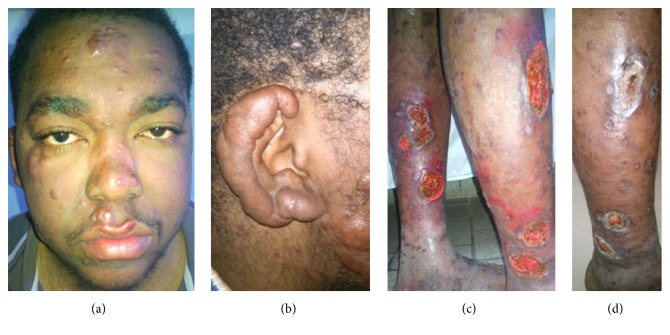
(a) Infiltrated papulonodular lesions on the face. (b) Infiltrated earlobe. (c) Multiple round and oval ulcers on legs. (d) Ulcers with sharply defined borders, margin erythema, and septic, purulent base.

Upon our examination, the patient appeared to be in fairly good condition without fever. His hemodynamic parameters were normal. He presented with multiple infiltrated erythematous papulonodular lesions on his face, nose, and upper lip (Figure 1(a)) and earlobe (Figure 1(b)). These painless lesions had symmetrically spread to his trunk, back, and limbs. His legs and right sole presented foul, round, and oval ulcers having a greatest diameter of 3.5 cm (Figure 1(c)) and with sharply defined borders, a discreet marginal erythema, and a dirty, purulent base masked by a colored antiseptic product (Figure 1(d)). Purplish stretch marks were visible on his flanks and underarms, as well as the presence of a buffalo neck. He had multiple enlarged lymph nodes, but palpable nerve trunk pain was absent, motricity was not deficient, and the blinking reflex appeared unaffected. However, we detected hypoesthesia on the outside edge of his left sole. Cardiopulmonary auscultation was normal.

**Figure 2:**
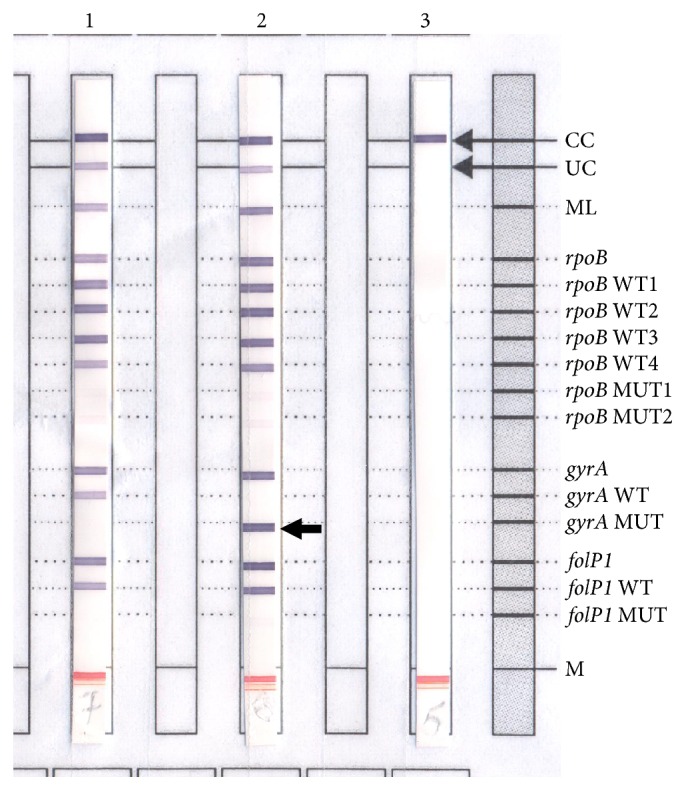
GenoType LepraeDR DNA strip test to assess drug resistance of* Mycobacterium leprae*. Lane 1, positive control using* M. leprae* wild-type strain with* rpoB*,* gyrA*, and* folP1* alleles. Lane 2,* M. leprae* strain of the case with* gyrA* mutation (A91V) and resistant to fluoroquinolone (arrow). Lane 3, negative control.

Biological tests showed marked inflammation as evidenced by accelerated erythrocyte sedimentation rate at 71 mm in the first hour, elevated C-reactive protein at 24 mg/L, and polyclonal hypergammaglobulinemia at 18 g/L on blood protein electrophoresis, despite a normal blood cell count. Blood sugar, creatinine, and liver enzymes were within normal ranges. HIV, hepatitis B, and hepatitis C serology proved negative. Chest radiography was normal. Arteriovenous Doppler ultrasound was performed to rule out vascular etiology for nonhealing ulcers. Histopathological findings were compatible with lepromatous leprosy, showing polymorphic infiltration of foamy lymphohistiocytic cells into the skin, often located around adnexal structures, but without Virchow's cells, nor any sign of vasculitis. Ziehl-Neelsen staining performed on a skin biopsy revealed a positive high score (5+) of acid-fast bacilli. Infection with* Mycobacterium leprae* was confirmed by PCR assay (GenoType® LepraeDR, Hain Lifescience). Quinolone resistance due to A91V mutation on the* gyrA* gene encoding the A subunit of DNA gyrase was identified by a technique previously described [4] (Figure 2). However, this strain was shown to be sensitive to rifampicin (no* rpoB* mutation) and dapsone (no* folP*1 mutation).

After 6 months of standard multidrug therapy regimen for multibacillary leprosy, a combination of rifampicin, clofazimine, and dapsone, most cutaneous lesions disappeared, and ulcers on the lower limbs healed. The bacillary index fell to 2+, although some infiltrated nodular lesions still remained. As of this date, no new cases have been reported among the persons who have been in close contact with the patient.

## 3. Discussion

Polar lepromatous leprosy is described as a highly contagious, multibacillary, cutaneous, and mucosal form of leprosy with frequent visceral involvement. This condition occurs when* M. leprae* multiplies and spreads into the blood due to the lack of the host's cellular immune response against the bacillus [1]. In addition, our patient presented apparent peripheral signs of corticoid impregnation, which may have explained the diffuse clinical features of lepromatous leprosy and the presence of longstanding nonhealing ulcers. The systemic administration of corticosteroid over a 4-year period without etiological treatment has worsened the lack of cellular immune response.

Typical lepromatous lesions are papulonodular “leproma,” which are symmetric in shape, and located on the face, particularly over the eyebrows, and on the earlobes and chin. Peripheral edema of the foot and hypoesthesia in the limbs may also be found. Our patient, examined at an advanced stage of the disease, presented all the signs mentioned. Even though Malagasy physicians practice medicine in a country where leprosy is endemic, very few typically recognize the cutaneous signs of the infection, thus leading to misdiagnosis and erroneous treatment. Unfortunately, leprosy often only refers to macular hypochromic and hypoesthetic lesions in daily practice.

Ulcers are an unusual clinical symptom of leprosy in the advanced stages of the disease. They are mainly due to a loss of sensation in connection with peripheral neuropathy and rarely due to a complication of erythema nodosum leprosy [5]. Lucio's phenomenon, a reaction that causes ulcers in leprosy, may be ruled out in our present study following histopathological findings. Lucio's phenomenon is a type of dermal leukocytoclastic vasculitis characterized by necrotic skin ulcers, preferentially affecting the lower extremities and usually associated with ongoing diffuse lepromatous leprosy [6].

Drug resistance in* M. leprae* has become a major public health concern worldwide [7]. It can be detected using molecular methods, as in our present case. Although rarely encountered [8, 9], quinolone-resistant* M. leprae* cases cannot be neglected, since quinolones are used as part of second-line treatment against drug-resistant strains, usually applied following secondary resistance observed in relapse cases [10]. The resistance mutation (A91V) is the one most commonly observed and here is likely a result of selection from continuous self-medicated doses of quinolones leading to secondary drug resistance. Limiting the overconsumption and supply of antibiotics by enforcing the requirement for medical prescription is the best approach for the prevention of atypical primary resistance.

In conclusion, there is a need to sustain leprosy expertise, even after its elimination at the global level. Clinicians must have access to up-to-date information on recognizing the symptoms of leprosy to avoid unnecessary delay in diagnosis and treatment. This will help reduce the risk of the disease from spreading. This approach can be explained in training programs to sensitize health workers throughout our country. Molecular diagnostic tests are now available and can be performed locally to accurately detect drug resistance to leprosy.
